# Exploring the Role of Sigma Receptors in the Treatment of Cancer: A Narrative Review

**DOI:** 10.7759/cureus.70946

**Published:** 2024-10-06

**Authors:** George Fotakopoulos, Charalabos Gatos, Vasiliki E Georgakopoulou, Grigorios Christodoulidis, Iraklis Kagkouras, Nikolaos Trakas, Nikolaos Foroglou

**Affiliations:** 1 Neurosurgery, Aristotle University of Thessaloniki, Thessaloniki, GRC; 2 Neurosurgery, General University Hospital of Larissa, Larissa, GRC; 3 Pathophysiology/Pulmonology, Laiko General Hospital, Athens, GRC; 4 Surgery, General University Hospital of Larissa, Larissa, GRC; 5 Surgery, Worcestershire Acute Hospitals, Worcester, GBR; 6 Biochemistry, Sismanoglio Hospital, Marousi, GRC; 7 Neurosurgery, Aristotle University of Thessaloniki, AHEPA University Hospital, Thessaloniki, GRC

**Keywords:** cancer, carcinogenesis, sigma receptors, s ligands, σ receptors

## Abstract

This study investigated the association of sigma receptors (SRs) and their selective ligands (because the molecular characteristics of the same SRs, particularly sigma-2 receptor {S2R}, are not completely clear) in carcinogenesis, their potential use as antitumor agents, and their great utility in tumor imaging. The ion channels and transporters enhance the cell's ability to adapt to the metabolic conditions encountered in the tumor tissue. The high expression of SRs in the proliferating cells compared with those at rest indicates that this is a significant clinical biomarker for determining the proliferative status of solid tumors using functional PET imaging techniques. The association of SRs in the pathophysiology of cancer cells is a result of the high concentration of S1R and S2R binding sites observed in various tumor cell lines and tissues. It would also be remarkable to determine if SRs are involved in metastasis and other metastatic cell behaviors such as adhesion, secretion, motility, and penetration. An absolute challenge for research in this field is to develop an integrated model that describes the molecular mechanisms of sigma receptors, incorporating their known biological and pathophysiological roles.

## Introduction and background

Sigma receptors (SRs) were first proposed in 1976 by Martin et al. as a distinct class of opioid receptors, based on the affinity of compounds like (-)-SKF-10,047 for these sites in opioid-dependent and non-dependent models [1]. The name "sigma" is derived from the initial letter of this model ligand. SRs are categorized into two subtypes as follows: sigma-1 receptor (S1R) and sigma-2 receptor (S2R), which are differentiated by their molecular weights, tissue distribution, and selective binding of various drugs [1]. The gene encoding S1R has been isolated from several species, including humans, and is characterized by its chromosomal location associated with psychiatric disorders [1]. Research into S2R, despite being less understood, suggests its significant role in the regulation of cancer cell proliferation and apoptosis [2-4].

The physiological relevance of SRs extends beyond the central nervous system to include widespread expression in various body tissues and their involvement in numerous cancers (mainly in the heart, liver, spleen, lung, kidney, adrenal gland, and gastrointestinal tract) [5]. S1R, in particular, has been identified in high concentrations in brain regions and peripheral organs, indicating its importance in both neurological and systemic functions [5,6]. On the other hand, studies have shown that S2R actively participates in cancer cell biology, influencing the dynamics of tumor growth and the effectiveness of cancer therapies. They have identified S2R as a potential marker of cellular proliferation in solid tumors, and they evaluated the proliferation of human breast cancer cell lines grown in vitro using Bromo-2'-deoxyuridine (BrdU) staining. However, they did not directly assess the ratio of proliferating to quiescent cells, known as the P:Q ratio [2,7]. Both receptors have potential clinical implications in oncology, particularly in the use of specific ligands for tumor imaging and targeted therapy [8,9]. This growing understanding of SR functions underscores their potential as pharmacological targets in various diseases, including cancer. In line with their broad modulatory role, S1 receptor ligands have been suggested for use in various therapeutic areas, including amnesic and cognitive deficits, depression and anxiety, schizophrenia, pain management, and mitigating certain effects of drugs of abuse, such as cocaine and methamphetamine [10].

Continued research into sigma receptors is important due to their complex role in cellular signaling pathways that concern cancer therapies. For instance, S1R interacts with various ion channels and signaling molecules, influencing mechanisms such as calcium signaling and protein phosphorylation, independent of traditional G-protein-coupled receptor pathways [11,12]. This interaction suggests a broader functional spectrum, impacting conditions ranging from neurodegeneration to cancer. The S2R subtype, while less characterized, shows promise in oncology as emerging studies reveal its role in modulating apoptosis and proliferation in cancer cells, potentially offering new avenues for therapeutic interventions [3,4]. The clinical significance of SRs is further highlighted by ongoing trials evaluating the efficacy of SR-targeted compounds, which could lead to novel treatments for difficult-to-treat cancers and other serious health conditions [9].

The scope of this review encompasses the biological roles, pharmacological significance, and therapeutic potential of SRs, specifically focusing on the sigma-1 (S1R) and sigma-2 (S2R) receptor subtypes. It aimed to elucidate the molecular characteristics, genetic backgrounds, and distinct physiological functions of each receptor subtype. Additionally, the review delves into the clinical implications of SRs in various diseases, particularly their emerging roles in cancer biology, such as influencing tumor growth, apoptosis, and therapeutic responsiveness. By examining both foundational research and recent advancements, the review seeks to highlight the potential of SR ligands as diagnostic tools and therapeutic agents in oncology and other medical fields.

## Review

Role of ion channels and S2R receptors in tumor aggressiveness and therapeutic targeting

The main cellular processes embed ion channels. That explains cancer's characteristics as follows: tumors often express ion channels and transporters that are absent in the surrounding healthy tissue. These channels and transporters help cells respond to the new metabolic conditions in the tumor tissue [13-15]. As a result, protein transporters are involved in the adaptation of tumor cells to a stressful environment. That gives them greater aggressiveness. S2R continues to be an important molecular target in the field of tumor biology. The high expression of this receptor in the proliferating cells, compared with those in the rest condition, indicates that it is a significant clinical biomarker for determining the proliferative status of solid tumors using the functional positron emission tomography (PET) imaging technique. The ability of S2R selective ligands to neutralize the volume through both apoptotic and non-apoptotic pathways suggests that this receptor could be a potential target for cancer chemotherapeutic agents. The statement that the putative binding site of S2R is the progesterone receptor membrane component 1 (PGRMC1) protein is important because it provides a strong scientific bridge between S2R and PGRMC1. It also makes available a wide range of potential small-molecule ligands to study the complex S2R/PPGRMC1 in solid tumors and cancer cells using a variety of experimental techniques [16].

S1R as chaperones related to carcinogenesis

The mitochondria-associated endoplasmatic membrane (MAM), where S1R is located, is in close contact with mitochondria at the endoplasmic reticulum (ER). At resting state, they are located within the ceramic and cholesterol-rich lipid microdomain, related to the ER region of 78 kDa GRP78 BiP (glucose-regulated protein, also known as binding immunoglobulin protein) [17,18]. Stress conditions damage the ER, cleaving S1R from the immunoglobulin heavy chain-binding protein (BiP) after connecting with inositol 1,4,5-trisphosphate receptor type 3 (IP3R3). This process increases cell survival by controlling Ca ion signaling between mitochondria and the ER [17].

Moreover, S1R travels to different cellular compartments and attaches itself to a variety of cellular proteins. SR agonists mimic the stressful breakdown of S1R from BiP and S1R rearrangement through activation, while SR ligands, classified as antagonists, block this process [17]. Thus, these results have led to the creation of a model where S1R keeps silent under normal circumstances but, in cases of disease, behaves as chaperones that bind the protein targets in favor of cell survival [16].

SR modulation from the ion channels

New research has found that S1R and ion channels interact at the molecular level, suggesting that ion channels are a large family of proteins that S1R targets as chaperones [19-22]. In recent decades, the main cellular process has incorporated ion channels, explaining the characteristics of cancer as follows: tumors often express ion channels, while the corresponding tissue lacks transporters [13-15]. It is believed that these channels and transporters enhance the cell's ability to adapt to the metabolic conditions encountered in the tumor tissue (low pH and PaO_2_, poor nutrient supply) [13-15]. Protein carriers play a crucial role in helping tumor cells adapt to stressful environments, which enhances their aggressiveness. Ion channels expressed in cancer cell lines of various types may significantly contribute to metastasis, a process that involves regulating cell growth and proliferation [23]. The observed upregulation of SR expression in tumor cells and tissue lines, functioning as auxiliary modules for certain ion channels, is particularly noteworthy given the growing body of evidence linking ion channels to both proliferation [23] and the metastatic activity of cancer cells [24]. A reduction in the amplitude of Kalium (potassium) ion (K+) channels has been correlated with the metastatic phenotype in prostate and breast cancers in humans, suggesting that this phenomenon could be relevant to the proposed connection between cancer development and S1R substances [22]. Besides proliferation, ion channel activity may influence cancer cell behavior through several mechanisms [25], including migration [26], apoptosis [27], adhesion, cytoskeletal organization, and secretion [28]. We still need to determine whether ion channels other than voltage-gated potassium (Kv) channels, such as voltage-gated sodium (Na⁺) channels, which are also composed of SR substances, contribute to the metastatic cascade [29]. If so, could this be a part of the cancer process?

SR modulation from the intracellular calcium ion (Ca^2+^)

Vilner and Bowen demonstrated that SR in neuroblastoma cells can use Ca^2+^ signals to trigger cellular effects [30]. The action of S1R specifically causes the rapid and transient release of ERs Ca^2+^, as demonstrated by the use of (1) SR-inactive substances, (2) S2R-selective agents like CB-64D, and (3) S1R-selective agents. In turn, intracellular Ca^2+^ formation may influence protein kinase C activity [31]. Indeed, at brain Indeed, S2R substances at mouse brain synapses regulate the activity of the dopamine transporter by activating protein kinase C [31]. Ca^2+^ signaling is very important for many cellular processes, so it can form a mechanism through which S2R medications and substances exert their effects on cancer cells [31].

SR modulation from the levels of sphingolipids

The sphingolipid levels in MCF-7, Adr-, and T47D breast cancer cell lines after applying S2R-specific agonists have been investigated to further understand the molecular mechanism by which S2R substances can cause morphological and apoptotic effects in various cancer cell lines [32]. The CB-184 causes a dose-dependent increase in ceramide levels and a simultaneous decrease in sphingomyelin in breast cancer MCF-7 and ADR-T47D cell lines [31]. N-phenyl piperidine, a non-specific competitor of SRs, reduces this effect. This effect suggests that S2R could utilize sphingolipid products to influence Ca^2+^ cell proliferation and survival signaling [32].

Signal transduction mechanism

S1R

Based on our current understanding of how K+ channels form S1R, we have identified a signal transduction mechanism for S1R that is (a) membrane-delimited [11,12]; (b) independent of G-protein coupling and protein phosphorylation [11,12]; (c) reconstituted in a heterologous system [2]; (d) does not require cytoplasmic factors [33]; and (e) requires close proximity between S1R and K+ channels [33], likely to form a stable macro-molecular complex [2]. Given the wide variety of functions referred to by S1R, it is likely a signaling mechanism of S1R, which comprises one or more intermediate signaling molecules (which are located on or into the plasma membrane) and not a direct interaction [11]. Furthermore, we have not yet determined the amino acid residues in Kv ion channels involved in this interaction (direct or indirect) with S1R [10]. Understanding these issues would shed more light on the S1R signaling mechanism.

S2R

According to a recent report, S2R and PGRMC1 are both intracellular and antagonize the same S2R ligands or PGRMC1 ligands, indicating a relationship between the relatively unknown S2R and the well-identified PGRMC1 protein [3]. Researchers have implicated the PGRMC1 protein in regulating the signal of steroids at P450 activation on antiapoptotic cesses, stimulating tumor growth, infiltration, and metastasis [3]. However, many results indicated that the S2R ligands, after caspase-3 activation, reduce the expression of p70S6K and 4E-binding protein 1 (4EBP1) (both are part of the signal pathway of mammalian target of rapamycin (mTOR), suppress the expression of cyclin D1, and induce poly (ADP-ribose) polymerase 1 (PARP 1) cleavage and deoxyribonucleic acid (DNA) fragmentation [3]. Researchers also found that other S2R ligands induce oxidative stress, mobilize intracellular Ca^2+^, and mediate Kv ion channels [3]. A more recent study found that S2R agonists greatly reduce the production of NF-kB, IL-2, TNF-a, and COX 2 in T lymphocytes.

In addition, there is strong evidence not only for the inhibitory effect of S2R ligands on p70S6K, 4EBP1, cyclin D1, PARP-1, and neutrophil gelatinase-associated lipocalin matrix metalloproteinase-9 (NGAL-MMP9) but also for the activation of caspase-3/7 [23]. It is also believed that S2R and/or PGRMC1 proteins have at least four signaling pathways in which they apply their regulatory properties [23]. The first pathway is the signaling of epidermal growth factor receptor (EGFR), promoting cell proliferation, while its inhibition leads to tumor cell apoptosis [23]. The second pathway interacts with the plasminogen activator inhibitor 1 RNA-binding protein (PAIR-BP1) and Insig-1, Scap, and sterol regulatory element-binding protein (SREBP), configuring the sterol composition and progesterone signaling [23]. The third pathway activates P450 and regulates lipid metabolism [23]. Finally, the fourth pathway may inhibit ER stress, while S2R ligand action exhibits the inhibitory role of S2R, causing the release of Ca^2+^, the consequent caspase activation, and the possible death of tumor cells [23]. EGFR activation and multiple signaling pathways, such as the phosphoinositide 3 kinase (PI3K)/Akt/mTOR pathway, the phospholipase C/pprotein kinase C(PLC/PKC)/NF-kB pathway, the trail RAS/RRAF/extracellular-signal-regulated kinase (ERK), and others, overexpress S2R in cancer, promoting cell proliferation and invasion [23]. Additionally, S2R can stimulate cancer cell growth by activating the P450 metabolic pathway, Insig-1, Scap, and SREBP pathways, and inhibiting caspase pathways. S2R binds to the ligand, interrupting or inhibiting the interaction with other effectors, thereby reversing their proliferative activity and promoting apoptosis [23].

Role of SR1 in cancer

Initial binding studies from the 1990s highlighted that sigma receptors are highly expressed in various human and rodent tumor cell lines, such as breast, lung, prostate, colon, melanoma, neuroblastoma, and glioma. However, these early studies struggled to distinguish between the two subtypes, S1R and S2R, due to the non-selectivity of the ligands used [34-36]. Later research utilizing an S1R-specific antibody confirmed a significant upregulation of S1R in certain cancer types, including breast, lung, and prostate cancers, while lower expression was observed in the corresponding normal tissues. Notably, increased S1R expression was associated with high metastatic potential, suggesting a link between receptor density and tumor aggressiveness [37].

Further immunohistochemical analysis of breast cancer patient samples revealed S1R positivity in 60% of invasive cancers, 41% of in situ cancers, and 75% of ductal hyperplasia, with only 33% of normal breast tissue showing S1R expression [38]. Additionally, scintigraphy using an S1R-specific ligand showed that this receptor is selectively retained in primary breast tumors, demonstrating its potential as a diagnostic marker [27]. Building on these findings, several studies have utilized S1R ligands to enhance the targeting and delivery of therapeutic nanoparticles to tumors, successfully improving drug delivery in models of melanoma, prostate, lung, and breast cancers [39,40].

Functional studies have explored the impact of sigma ligands on cancer cell behavior, revealing that treatment with these ligands can induce significant morphological changes (cell rounding and detachment) and inhibit growth in glioma, breast, colon, and melanoma cell lines. The moderately selective S1R ligand rimcazole, for instance, has been shown to trigger caspase-dependent apoptosis in breast and colon cancer cells both in vitro and in vivo. The apoptotic effect of rimcazole is linked to S1R-dependent pathways, involving a complex interplay of calcium signaling and inhibition of the phosphatidylinositol 3-kinase (PI3K) pathway. Notably, the presence of S1R agonists, such as pentazocine or SKF10,047, can block rimcazole-induced apoptosis. This suggests that S1R is likely in an activated state in cancer cells, promoting cell survival [41,42].

The role of S1R in cell-matrix interactions has also been highlighted, particularly in breast cancer cells, where S1R associates with β1 integrin in lipid rafts. This association appears critical for cell adhesion to extracellular matrix components such as fibronectin and vitronectin. Silencing S1R disrupts this interaction, reducing cell adhesion and potentially affecting tumor invasiveness. Intriguingly, the S1R agonist SKF10,047 reduces β1 integrin levels in lipid rafts and diminishes adhesion, similar to the effects observed when depleting membrane cholesterol, suggesting that S1R might modulate cell adhesion through its influence on lipid raft composition [43].

Recently, mutations in S1R have been linked to neurodegenerative conditions, such as amyotrophic lateral sclerosis (ALS) and frontotemporal lobar degeneration. This discovery raises questions about whether similar mutations might be present in cancer tissues, potentially contributing to the functional variability of S1R in different pathologies. The overarching hypothesis suggests that S1R acts as a sterol-dependent, stress-activated chaperone that modulates lipid raft formation in both the endoplasmic reticulum and plasma membrane. This chaperoning activity could dynamically alter cancer cell behavior in response to the hostile microenvironment typical of tumors, which includes hypoxia, nutrient deprivation, and oxidative stress. The precise role of S1R in cancer remains complex and context-dependent, potentially involving a diverse array of client proteins, particularly ion channels, that modulate cancer cell physiology in a tumor-specific manner [20].

SR ligands as anticancer agents

N-cyclohexyl-N-ethyl-3-(3-chloro-4-cyclohexylphenyl) propen-2-ylamine hydrochloride (SR31747A) is a peripheral selective SR ligand. In mouse xenografts, this molecule has potent in vitro and in vivo anti-proliferative activity against cell lines of different tumor models [9]. Based on these findings, researchers are currently testing SR31747A for prostate cancer as a potential anticancer agent. Selective S2R ligands are known to regulate cancer cell growth. S2R ligands may represent a new molecular platform for the treatment of pancreatic and other cancers. It is believed that S2R agonists inhibit the proliferation of cancer cells and induce apoptosis, while S2R antagonists promote the survival of tumor cells. In addition, there are studies that have shown for the first time that SR ligands affect the morphology of C6 glioma cells, inhibit cell division, and cause cell death. Cancer cells' affinity for the S2R correlates with their degree of growth inhibition, which appears to depend on both their concentration and time of action [2,30]. Kashiwagi et al. discovered that S2R ligands turn on caspase-3 and cause cancer cells to die in a way that depends on their concentration and time, but pentazocine, an S1R ligand, does not have this effect [44]. S2R agonists CB-64D and CB-184 inhibit cancer cell growth in both drug-sensitive MCF-7 and drug-resistant tumor cell lines, such as MCF7/AADR, SKBR3, and T47D, and induce apoptosis in these tumor cells in a dose-dependent manner [45]. Siramesine, one of the most potent selective S2R ligands, has potential antitumor activity against various tumor cell lines as follows: (1) in breast tumors, (2) in neck tumors, (3) in lung tumors, (4) in prostate tumors, (5) in fibrosarcoma, and (6) in breast cancer grafts in mice [46,47]. Recent research has revealed that siramesine triggers caspase-3-dependent apoptosis in epithelial cells, suggesting the potential application of S2R ligands in the treatment or prevention of posterior capsular opacification [45]. In addition, this substance effectively induces apoptosis (in vitro and in vivo) at the WEHI-S cells (of mice with fibrosarcoma) and MCF-7 cells in a dose-dependent manner [45]. Oxidative stress and massive lysosomal permeability induce these antitumor effects, independent of caspases, p53, Bcl-2, and cytochrome c [45]. Researchers have established selective S2R ligands and SW43 to trigger cancer cell apoptosis by increasing the permeability of the lysosomal membrane and cellular oxidant stress, but they do not require the activation of caspase-3 [48]. Other S2R ligands, like WC26, SV119, and RHM-138, seem to work against tumors by making caspase-3 more active (the ZAVD-FMK stops the depression of DNA fragmentation and cytotoxicity), starting autophagy, and lowering the activity of the mTOR pathway [49]. Therefore, multiple signaling pathways likely comprise the mechanism involved in S2R ligand-induced apoptosis [3].

S2R and various cancers

The S2R receptor may induce apoptosis, specifically in pancreatic cancer, by increasing the effect of the standard treatments [49]. The discovery that S2R ligands accumulate in malignancies and increase their density in proliferating cancer cells makes the use of PET or single photon emission computed tomography (SPECT) imaging with this class of molecules possible [8]. After photoaffinity binding to a new binder of WC-21 and the known ligand of PGRMC1, AG-205, researchers identified PGRMC1 as the most likely binding site of the S2R [49,50]. This significant discovery validates the overexpression of PGRMC1 and S2R in pancreatic cancer, as previous research has demonstrated that small-molecule inhibitors can target PGRMC1 [51]. Similarly, S2R has been targeted with small-molecule inhibitors in the treatment of pancreatic cancer [49,51]. Traditionally, PGRMC1 has been studied for its overexpression and sensitivity to progesterone in ovarian and breast cancers [51,52]. Progesterone exhibits anti-apoptotic activity in ovarian cancer, but cells depleted of PGRMC1 showed increased apoptosis when treated with progesterone [53]. Recently, PGRMC1 overexpression was identified in both the serum and tumors of patients with squamous cell carcinoma and lung adenocarcinoma [20]. PGRMC1 expression has been shown to modulate ovarian cancer's sensitivity to chemotherapy and is linked to alpha estrogen receptors and hypoxia in breast cancer [51,54,55].

S2R was first identified through receptor binding studies in PC12 cells, a cell line derived from pheochromocytoma in the adrenal glands of mice, which revealed a high density of both S2R and S1R in various human and mouse tumor cells [56]. Generally, the density of S2R in tumor cell lines is higher than that of S1R, with the exception of the prostate cancer cell line LNCaP.FGC and THP-1 in leukemia [56]. One key measure of growth in solid tumors is proliferative status (PS), defined as the ratio of proliferating cells (P) to quiescent cells (Q), which may be caused by nutrient deprivation or hypoxia (PS = P/Q) [57]. A similar measure, the growth fraction (GF), calculates the ratio of P cells in a tumor relative to the total number of P and Q cells (GF = P/P + Q) [58]. Research has shown that S2R is a useful biomarker for assessing the PS and GF of solid tumors through imaging techniques such as PET and SPECT [58]. Using the standard diploid cell line of adenocarcinoma in mice, researchers demonstrated that S2R concentrations are higher in proliferating cells (66P) than in quiescent cells (66Q) [58]. The relatively high concentration of S2R in Q cells suggests that S2R-selective radiosensitizers can distinguish between tumors in a quiescent state and normal tissues that are either senescent or quiescent [58]. Additionally, because most chemotherapeutic agents target P cells rather than Q cells, a high concentration of S2R in Q cells could help identify chemotherapy-resistant areas of a solid tumor [58].

SR substances as assistants in tumor imaging

**Figure 1:**
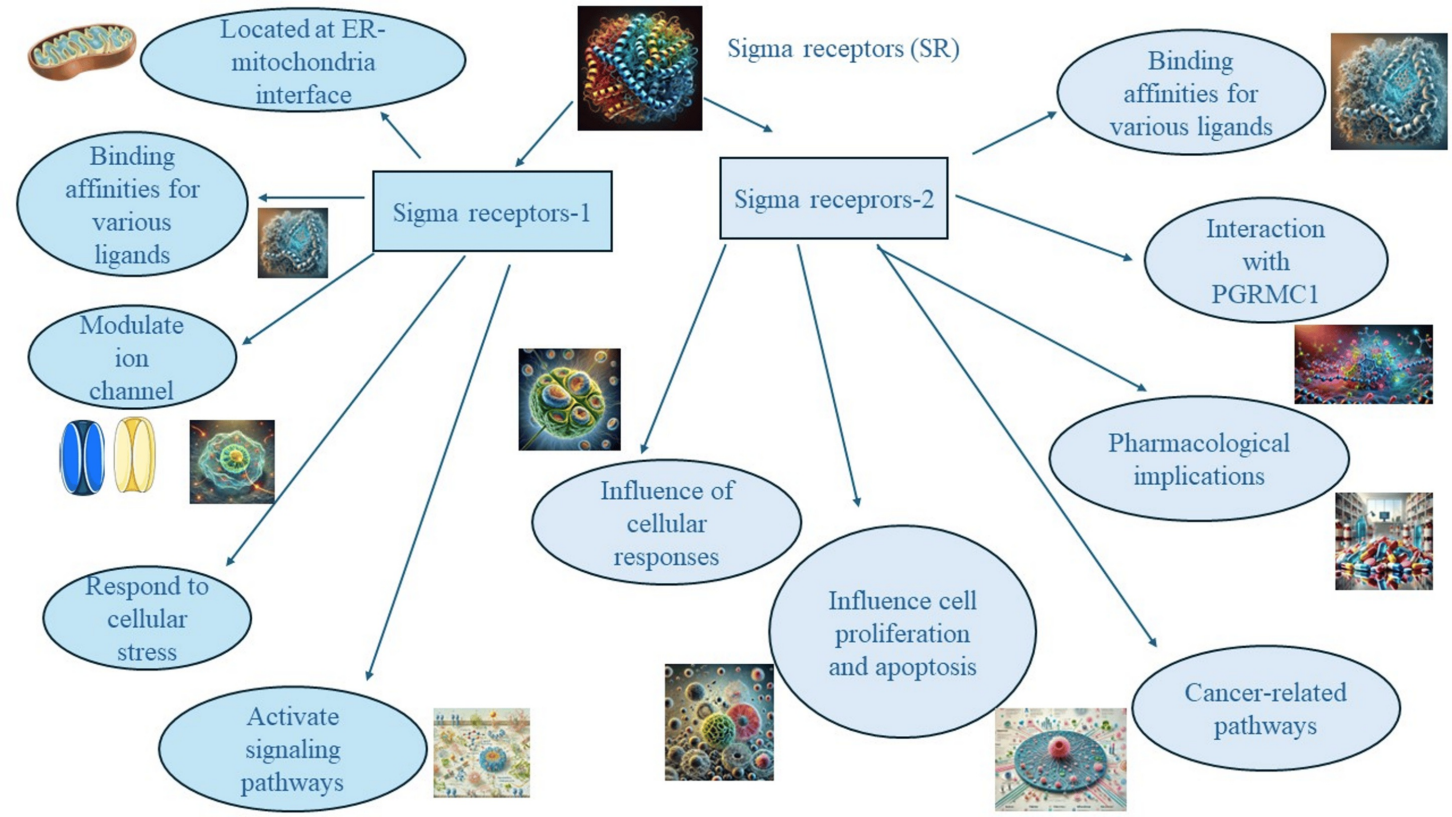
Schematic diagram of molecular interactions focusing on how sigma receptors operate in the context of cancer. The image is created by the authors of this study.

The high concentrations of S1R and S2R binding sites in tumor cell lines and tissues are an indication of their involvement in cancer cell pathophysiology and could have diagnostic potential in tumor imaging [59]. Radiolabeled SR substances have been tested in many preclinical studies to see how well they work as tumor imaging agents in melanoma [59], breast cancer [60], prostate cancer [61], and non-small cell lung cancer tumor models in mice [59]. Overall, these observations suggest that SR substances could be effective ligands for imaging the tumor in conjunction with techniques such as PET or SPECT [58,61,62]. Most of these SR substances are non-selective for S1R and S2R, but recent research has found S2R-selective agents particularly promising in this field [63]. Kashiwagi et al. conducted a study on the selective S2R ligand WC26, discovering its potential as a compound for tumor diagnostic imaging and as an anticancer agent [44]. More specifically, the S2R ligand demonstrated toxicity toward tumor cells while exhibiting little to no toxicity to normal tissues, as evidenced by measuring the percentage of apoptosis (via caspase-3 activity) in various tissue types, along with biological analysis of plasma, immunohistochemistry, and observations of normal behavioral patterns [44]. The SV119 S2R ligand also inhibited not only the propagation of malignancy cells in vitro but also tumor growth in mice while enhancing the apoptotic activity of gentamycin, with no evidence of systemic toxicity [44]. Figure 1 illustrates the molecular interactions focusing on how sigma receptors operate in the context of cancer.

New developments of sigma receptor action mechanism in carcinogenesis and perspectives for cancer treatment

Recent investigations have revealed that most cancer cells primarily use aerobic glycolysis, rather than mitochondrial oxidative phosphorylation, a phenomenon known as "Warburg," to generate the power required for the appearance of adenosine triphosphate (ATP) in their cellular processes [64]. In addition, DeBerardinis et al. proved that glycolysis, oxidative phosphorylation, the pentose phosphate pathway, and glutamine metabolism are interconnected proliferating cells to produce lipids and nucleic acids required for nucleotide and lipid composition [64]. These processes mean that cancer cells don't need to make a lot of ATP in their mitochondria. Instead, they use glutamine breakdown in the tricarboxylic acid cycle (TCA) to make lipoid and nucleic acids [64]. In the cancer cells, the carboxyl (-COOH) terminus of the S2R agonist reacts with both P-glycoprotein (P-gp) and the S2R. These combined activities activate an aimless ATP cycle and produce reactive oxygen species (ROS) [65]. Researchers are studying this phenomenon, known as collateral sensitivity, in tumors resistant to many drugs, and the complex substrates S2R agonists and P-gp offer promising therapy for these tumors [65]. These mechanisms represent an efficient process for the production of the high biomass quantity required from the rapidly proliferating cancer cells, particularly phospholipids, glycolipids, and cholesterol, which form the cell and organelle membranes [65]. In this context, SR is involved in regulating sphingomyelin, glucosylceramide, and cholesterol [66]. We could assume that overexpression of SR in cancer cells is beneficial, especially for the high production of lipids needed to form the plasma membrane in rapidly proliferating cancer cells.

In addition, deregulation of the normal composition of lipids by S1R ligands in highly proliferating tumor cells would interfere with cell division, as proposed, and so S1R ligands (agonists or antagonists) would strongly alter the mitotic process in tumor cell gliomas, regardless of S1R level expression [65]. Conversely, weak proliferating cells, including granulomatous neurons of the cerebellum, even those that are rich in S1R positions, show no sensitivity to the S1R antagonist 1-(4-iodophenyl)-3-(2-adamantyl) guanidine (IPAG), unlike the highly proliferating tumor cells [62].

Concerning the cancer treatment, assumptions are made that the high levels of SR ligands may induce a rapidly decreased expression of the modulator of Ras homolog gene (Rho) family G proteins (Rho GDI), which allows the activation of Rho guanosine-triphosphate (GTP) as required to transfer complexes S1R and inositol 1,4,5-trisphosphate receptor type 3 (IP3R3) and results in inhibition of ion flux, especially of Ca^2+^, and simultaneous deregulation of the ceramide biosynthesis pathway [62]. These two effects contribute to the disruption of the synthesis of the lipids, the major components of the membrane (such as phospholipids, glycolipids, and cholesterol), leading to disruption of mitosis in cancer cells overexpressing the SR [62]. Under ER-stressor factors, tumor cells overexpress S1R sensitivity, potentially explaining these effects.

## Conclusions

In conclusion, SRs are involved in various cellular functions that highlight their pathological significance. The connection between SRs and cancer cell pathophysiology is attributed to the high density of S1R and S2R binding sites observed in numerous tumor cell lines and tissues. As a result, researchers have proposed that SR substances could serve as promising agents for tumor imaging. Investigations into S2R-targeting drugs have explored their ability to inhibit cancer cell proliferation through mechanisms such as apoptosis, intracellular Ca^2+^ regulation, and sphingolipid pathways, potentially leading to the development of new cancer therapeutic agents. It is suggested that increased S2R expression may play a critical role in the transformation of a healthy cell into a malignant one, though more comprehensive studies are needed.

It would also be valuable to determine whether SRs are involved in metastasis - a multi-step process that includes cell proliferation - and in other metastatic behaviors such as adhesion, secretion, motility, and tissue invasion. The proposed molecular model of S1R is instrumental in understanding their cellular functions and may aid in elucidating the molecular basis of S2R. Additionally, the nature of endogenous SR ligands remains a crucial area for future research. It is highly likely that SRs do not function as "classic" ligand receptors. A significant challenge in this field is the development of an integrated model that describes the molecular mechanisms of SRs, incorporating their known biological and pathological roles. A thorough molecular investigation of SR activity is essential to better understand their role in cancer biology and could potentially lead to the discovery or design of new SR-targeting agents that may be used as diagnostic and/or therapeutic tools in cancer management.
